# Naturally occurring nanoparticles from English ivy: an alternative to metal-based nanoparticles for UV protection

**DOI:** 10.1186/1477-3155-8-12

**Published:** 2010-06-09

**Authors:** Lijin Xia, Scott C Lenaghan, Mingjun Zhang, Zhili Zhang, Quanshui Li

**Affiliations:** 1 Department of Mechanical, Aerospace and Biomedical Engineering, University of Tennessee, Knoxville, TN, 37996, USA

## Abstract

**Background:**

Over the last decade safety concerns have arisen about the use of metal-based nanoparticles in the cosmetics field. Metal-based nanoparticles have been linked to both environmental and animal toxicity in a variety of studies. Perhaps the greatest concern involves the large amounts of TiO_2 _nanoparticles that are used in commercial sunscreens. As an alternative to using these potentially hazardous metal-based nanoparticles, we have isolated organic nanoparticles from English ivy (*Hedera helix*). In this study, ivy nanoparticles were evaluated for their potential use in sunscreens based on four criteria: 1) ability to absorb and scatter ultraviolet light, 2) toxicity to mammalian cells, 3) biodegradability, and 4) potential for diffusion through skin.

**Results:**

Purified ivy nanoparticles were first tested for their UV protective effects using a standard spectrophotometric assay. Next the cell toxicity of the ivy nanoparticles was compared to TiO_2 _nanoparticles using HeLa cells. The biodegradability of these nanoparticles was also determined through several digestion techniques. Finally, a mathematical model was developed to determine the potential for ivy nanoparticles to penetrate through human skin. The results indicated that the ivy nanoparticles were more efficient in blocking UV light, less toxic to mammalian cells, easily biodegradable, and had a limited potential to penetrate through human skin. When compared to TiO_2 _nanoparticles, the ivy nanoparticles showed decreased cell toxicity, and were easily degradable, indicating that they provided a safer alternative to these nanoparticles.

**Conclusions:**

With the data collected from this study, we have demonstrated the great potential of ivy nanoparticles as a sunscreen protective agent, and their increased safety over commonly used metal oxide nanoparticles.

## Background

Ultraviolet (UV) radiation is highly energetic electromagnetic radiation from light waves below that of the visible light spectrum. The wavelengths for UV radiation range from 100-400 nm, including UV-A (315-400 nm), UV-B (280-315 nm), and UV-C (100-280 nm) [1]. While the earth's ozone layer blocks 98.7% of UV radiation from penetrating through the atmosphere, a small percentage of UV, comprising UV-A and some UV-B, can still reach the planet, which can cause harmful effects to humans [2]. UV-C does not typically reach the surface of the planet, but due to its ability to cause DNA damage it is often used as a model for UV study in the laboratory. Depending on the time of exposure to sunlight, the harmful UV-A/UV-B effects include immediate distresses like blistering sunburns, and long term problems like skin cancer, melanoma, cataracts, and immune suppression [3,4]. The underlying mechanism for UV-A damage involves oxidative stress and protein denaturation, while short wavelength UV-B radiation causes predominantly DNA damage in the form of pyrimidine dimers and 6-4 photoproducts [5]. UV radiation induced DNA mutation is one of the leading causes of skin cancer, with more than one million cases diagnosed annually resulting in 11,590 deaths in the U.S. [6].

The demand for skin protection agents against the harmful influence of UV solar radiation has become increasingly important in light of the depletion of the ozone layer [7,8]. Sunscreens, which work by combining organic and inorganic ingredients to reflect, scatter or absorb UV radiation, provide significant protection against the damage from solar UV. Early sunscreens developed with inorganic UV filters, such as titanium dioxide (TiO_2_) and zinc oxide (ZnO) particles, were often opaque giving the skin a white tinge, which made them unappealing to consumers [9]. With enhanced UV protection and low opacity, nanosize metal oxide particles have been introduced into cosmetics products in recent years and thousands of tons of nanomaterials are currently applied onto the faces and hands of hundreds of millions of people every year [10]. With increased popularity, the safety of these metal-based nanoparticles and potential toxicity is under significant debate. Many studies indicated that when applied to skin for less than 8 hours, inorganic nanoparticle filters do not penetrate through the stratum corneum (SC) layer of the skin [11-14]. However, these studies typically examine the effects of nanoparticles greater than 20 nm, and always use healthy skin samples. Studies evaluating the penetration of ultrafine nanoparticles found that TiO_2_, maghemite, and iron nanoparticles less than 15 nm are capable of penetrating through the SC [15,16]. Other studies have also observed penetration of 4 nm and 60 nm TiO_2 _particles through healthy skin in hairless mice after prolonged exposure from 30-60 days [17]. This penetration leads to increased aging of skin, pathological effects in the liver, and particle accumulation in the brain. Studies like these have raised significant concerns about the prolonged use of these metal oxide nanoparticles for cosmetic applications which lead to investigation of alternative organic filters.

The properties of materials at the nanoscale differ significantly from those at a larger scale, and safety claims by cosmetics manufacturers based on their bulk properties pose great risk without proper federal regulation of their applications [18]. When decreasing size to the nanoscale, materials alter many of their physical and chemical properties, including but not limited to color, solubility, material strength, electrical conductivity, magnetic behavior, mobility (within the environment and within the human body), chemical and biological activities [19]. The increased surface to volume ratio also enhances chemical activity, which can result in the increased production of reactive oxygen species (ROS) [20]. ROS production, which has been found in metal oxide nanoparticles, carbon nanotubes, and fullerenes, is the leading force of oxidative stress, inflammation, and consequent damage to DNA, proteins and membranes [20]. Further concern for these nanomaterials in applications is their photoactivity when exposed to UV light, which results in greater ROS and free radical production [21]. TiO_2 _nanoparticles have been shown to cause far greater damage to DNA than does TiO_2_of larger particle size [22]. While 500 nm TiO_2 _particles have some ability to cause DNA strand breakage, 20 nm TiO_2 _nanoparticles are capable of causing complete destruction of super-coiled DNA, as demonstrated in a plasmid DNA assay, even at lower doses and without exposure to UV. In addition to the increased potential for DNA damage from engineered metal oxide nanoparticles, another concern for their application in cosmetics is the potential for inhalation, ingestion, and penetration through the skin. Once in the blood stream, nanomaterials can be circulated inside the body and are taken up by organs and tissues such as the brain, liver, spleen, kidney, heart, bone marrow, and nervous system [19]. With their stability, the damage of these nanoparticles to human tissues and organs can occur through a traditional ROS pathway, or through accumulation that can impair their normal functions. *In vitro *studies on BRL 3A rat liver cells exposed to 100-250 μg/ml of Fe_3_O_4_, Al, MoO_3 _and TiO_2 _nanoparticles revealed significant damage from ROS in these cells [23]. Carbon nanotubes have also been shown to be toxic to kidney cells and inhibit cell growth [24]. The stability of nanomaterials in the environment has also been linked to brain damage and mortality in several aquatic species [25,26].

Due to the potential toxicity associated with prolonged use of metal oxide nanoparticle sunscreens, it is crucial to search for alternative ingredients that are non-toxic and effective at blocking UV. It is highly expected that these ingredients should be biodegradable, and less toxic to mammalian cells than metal oxide nanoparticles. The recent discovery of ivy and other naturally occurring nanoparticles provides a promising alternative to engineered metal oxide nanomaterials for cosmetics applications [27]. To explore the possibility of using ivy nanoparticles for sunscreen, the UV protection properties of ivy nanoparticles were investigated in this study. In addition, skin penetration, cytotoxicity, and environmental risks of ivy nanoparticles have also been investigated in this study.

## Results and Discussion

### Ivy nanoparticle isolation and topographic characterization

**Figure 1:**
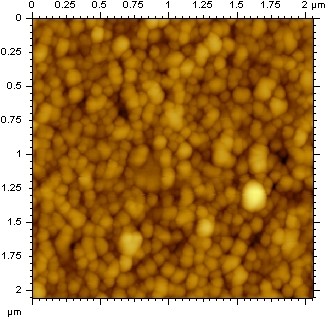
**AFM characterization of ivy nanoparticles**. The image shows a 2.05 × 2.05 um AFM view of isolated nanoparticles from cultured ivy rootlets, which indicated a high abundance of nanoparticles with an average diameter of 65.3 ± 8.04 nm.

The first stage in assessing the UV protective abilities of the nanoparticles required isolation and purification of the nanoparticles from the aerial rootlets. In order to facilitate easier collection of rootlets, we developed a tissue culture method for the aerial rootlets that allowed them to grow on culture plates in a nutrient agar. Another advantage of this technique is that the aerial rootlets grown in this culture system are sterile, and free from any environmental contaminants. Atomic force microscopy (AFM) studies were conducted on the cultured rootlets, and their wild counterparts. Similar nanoparticles were observed as originally discovered in wild ivy [27]. In order to generate bulk preparations of nanoparticles, we harvested the aerial rootlets from culture plates and homogenized them in a microfuge tube with heat sterilized forceps. To remove the large debris, the homogenate was centrifuged at 9,000 × g for 10 minutes. This separated large cellular debris from the much smaller nanoparticles. The supernatant was then filtered through a 200 nm filter and dialyzed to remove compounds with a molecular weight less than 12,000 daltons. The dialyzed solution was then loaded to a BioSep-SEC-S 4000 column developed by Phenomenex (Phoenix, AZ), with a flow rate of 0.5 ml/min, and all fractions were collected. These fractions were then analyzed by AFM for the existence of expected ivy nanoparticles. From Figure 1, we could observe that the isolated ivy nanoparticles had a diameter of 65.3 ± 8.04 nm, based on measurements of 30 randomly counted nanoparticles that were not dominated by other particles. Large agglomerates of nanoparticles ranging from 100-200 nm were ignored from the particle size determination.

### UV extinction of ivy nanoparticles

To demonstrate the ability of ivy nanoparticles for UV protection, we measured the optical extinction spectra of these nanoparticles using an ultraviolet and visible wavelength (UV-Vis) spectrophotometer. From the experimental data, we observed that the ivy nanoparticles, at a concentration of 4.92 μg/ml, had significant extinction in the ultraviolet region, while having little extinction at the visible and near infrared regions. This indicated that ivy nanoparticles would effectively block UV radiation without the opacity observed in other metal-based nanoparticles.

**Figure 2:**
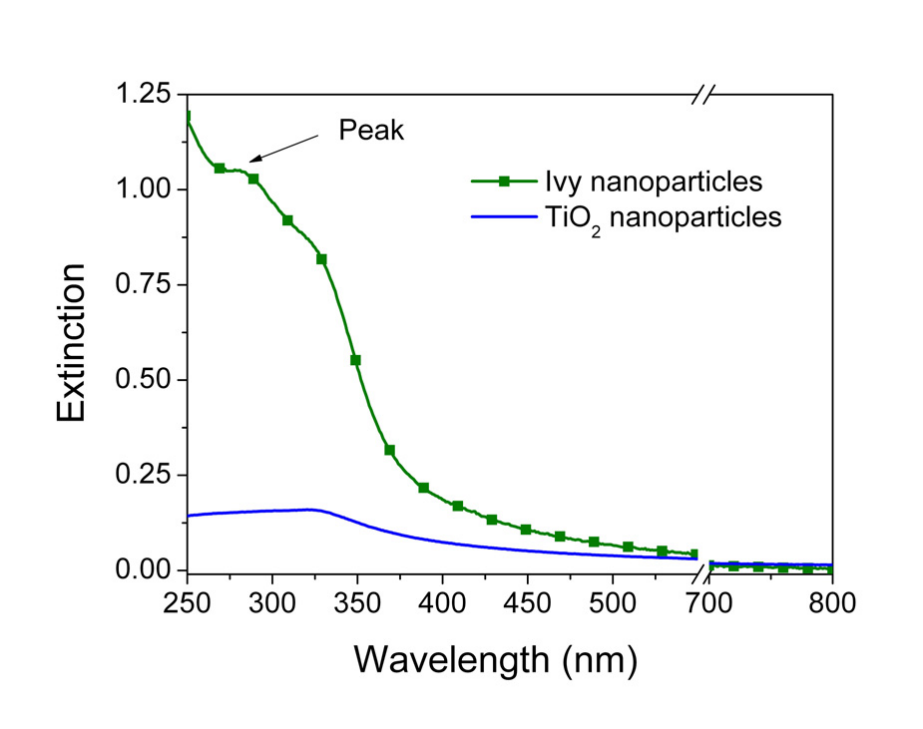
**UV extinction spectra**. Spectral profiles represent the UV extinction of the 4.92 μg/ml ivy and TiO_2 _nanoparticles. The green line corresponds to the ivy nanoparticles, while the blue line corresponds to the TiO_2 _nanoparticles.

Comparison of the UV blockage with TiO_2 _nanoparticles at the same concentration indicated that the total extinction of the ivy nanoparticles from 280 nm to 400 nm was much better than that of the TiO_2 _nanoparticles (Figure 2). The extinction of the ivy nanoparticles decreased sharply after the UV region, which makes ivy nanoparticles more effective in the UV-A/UV-B region and gives them high transmittance in the visible region making them virtually "invisible".

Our previous studies have confirmed that ivy nanoparticles are organic and have unique adhesive properties [27]. The adhesive effect of these nanoparticles will allow the ivy nanoparticles to remain on the skin for a longer period of time, and thus enhance their UV protective effect. The combination of these unique factors makes the ivy nanoparticles an appealing candidate for the development of a novel sunscreen product.

### Cytotoxicity

Although nanoparticles greater than 20 nm in diameter have not been reported to permeate through human skin, this data was obtained using healthy individuals in an optimal setting [15]. In specific cases, the skin structure can be changed to allow the penetration of large particles into the blood system, which has been demonstrated by the ability of 1,000 nm particles to access the dermis when intact skin is flexed [28]. More frequently, however, when skin is damaged, as in the case of people with sunburn, blemished skin, frequent shaving, or massages, there will be an increased risk of penetration [29-31]. A recent report by the US-based Environmental Working Group on the health risks of commercially available cosmetics and personal care products found that more than half of all cosmetics contained ingredients that act as "penetration enhancers" [32]. This raises further concerns for the safety of applied nanoparticles for personal care and cosmetics, since these agents will presumably increase the penetration potential of nanoparticles. As such, the cytotoxicity of nanoparticles should be thoroughly tested before their application in sunscreens.

**Figure 3:**
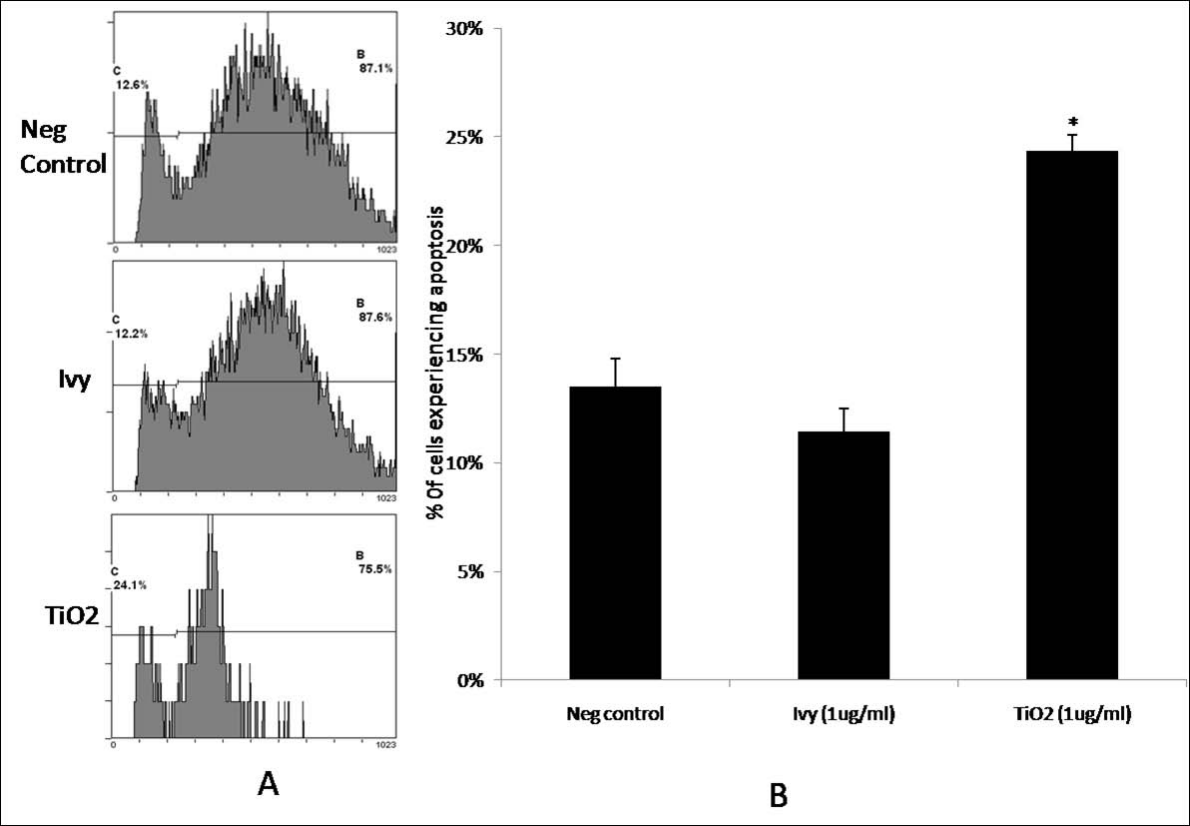
**Cytotoxicity analysis**. HeLa cells were incubated with or without nanoparticles for 24 hours and stained with propidium iodide. The cell apoptosis was then determined by detection of fluorescence using flow cytometry. A) Representative flow cytometry plots for each of the three samples: negative control (Neg control), ivy nanoparticle (Ivy), TiO_2 _nanoparticle (TiO_2_). B) Cells experiencing apoptosis in the three samples. Each point represents an average of 3 samples from one of three experiments. * denotes significant difference based on Student's *t *test (p < 0.05).

Due to the increased toxicity associated with internalized nanoparticles, as mentioned earlier, we have chosen to examine the toxicity of a mammalian endothelial cell line, HeLa cells. HeLa cells are commonly used for testing the toxicity and trafficking of nanoparticles [33-35]. In the experimental study, we incubated 1 μg/ml ivy nanoparticles with HeLa cells for 24 hours to test the cytotoxicity of these ivy nanoparticles. The toxicity was determined using propidium iodide staining and was examined by flow cytometry. We observed no toxicity compared to the control cells upon incubation with the ivy nanoparticles. However, in the same study, the same concentration of TiO_2 _nanoparticles exhibited significant toxicity to HeLa cells. In a standard flow cytometry experiment (Figure 3A), Gate C in each plot was defined for the cell population with less DNA, and thus represented the cells experiencing apoptosis. Gate B from the plots was the HeLa cells with more DNA, which indicated replicating cells at differing growth stages. Statistical analysis concluded that there was no significant difference in the percentage of cells experiencing apoptosis between the control cell population (13.5% ± 1.27%) and cells incubated with ivy nanoparticles (11.5% ± 1.06%) (Figure 3B). However, in the cells incubated with TiO_2_, 24.3% ± 0.7% of cells were experiencing apoptosis, which was significantly higher than the control cell population (p = 0.011) and the cells incubated with ivy nanoparticles (p = 0.007).

### Degradation

Although we addressed the cytotoxicity of ivy nanoparticles in the HeLa cell line, the possibility of these ivy nanoparticles exhibiting toxicity in the body may still exist. There have been observations with gold-dendrimer nanoparticles accumulating in the liver that might damage normal liver function [36]. To address this concern for ivy nanoparticles, we tested the ability of the nanoparticles to be degraded should they pass through the skin or mucous membranes. If the ivy nanoparticles were degradable, then they would be digested after their penetration through the skin and lose their normal nano-structure and thus any toxicity based on the nano-morphology of the particles. The degradability of nanomaterial is also beneficial to the environment when considering reports that nanomaterials have been linked to damage in fish, mortality in water fleas, and have bactericidal properties that can impact ecosystems [14,19]. Thus, the biodegradability of these ivy nanoparticles was also investigated in this study.

**Figure 4:**
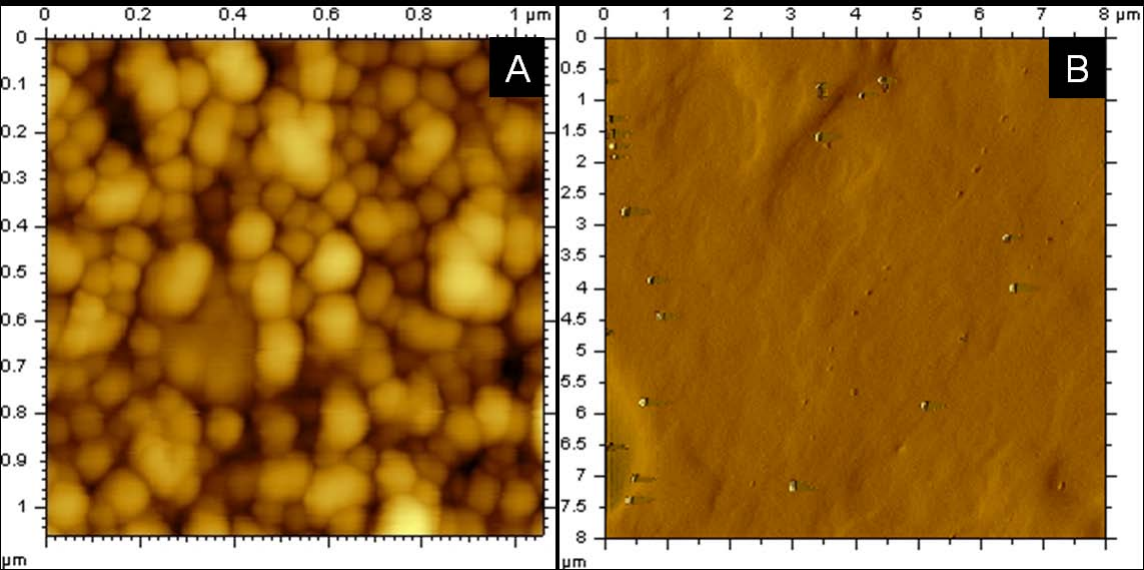
**Biodegradability of ivy nanoparticles**. The isolated ivy nanoparticles were incubated without (A) or with (B) Proteinase K at 37°C for 30 min. The structure of the samples was then analysed with AFM using tapping mode.

Our experimental studies indicated that at temperatures from 4-37°C, the ivy nanoparticles were stable and could be readily imaged by AFM. In addition, sonication from 5-9 W was not effective at destroying the particle structure, but did serve to disperse the particles and prevent the formation of large agglomerates. Incubation of the ivy nanoparticles in RPMI, a common cell culture media, at 37°C for up to 24 hours did not result in digestion of the nanoparticles as assessed by AFM. To test the ability for the particles to be broken down by enzymatic digestion, Proteinase K was used in an attempt to digest the nanoparticles. Digestion was carried out from 15 minutes to 4 hours, to determine if the extent of incubation affected the digestion of the particles. After incubation with Proteinase K for 30 min, it was no longer possible to image the nanoparticles with AFM. As shown in Figure 4, after enzymatic digestion, the ivy nanoparticles were degraded and lost their normal structure. This enzymatic digestion by a common proteinase could further reduce the risk to the environment and human tissues and organs. This gives organic nanoparticles a definitive advantage over metal oxide nanoparticles, since these particles resist breakdown by biological organisms and remain in the body or environment for prolonged periods of time. We would like to point out that the above nanoparticles used for the Proteinase K digestion were collected based on size which also matched well with the UV 280 nm detection. As expected, they have been totally degraded. However, we could not eliminate the possibility that there might be other type/size of nanoparticles that had not been detected by UV detector, and as a result, were not collected for the Proteinase K digestion. The long-term objective of this study is to propose protein-based ivy nanoparticles for UV protection.

### Skin penetration

Another major concern with cosmetic nanoparticles is their probability to penetrate through the skin into the circulatory system [37,38]. The development of proper markers for the detection of ivy nanoparticles in the skin takes considerable time, however, a simple mathematical modeling and computational approach may allow rapid analysis for the potential of ivy nanoparticles to penetrate though skin.

Skin structure, is composed of the protective outer SC layer and a viable epidermis and dermis layer with other accessory glands [39]. The penetration of particles in the skin can occur through pilosebaceous pores (diameter: 10-70 μm), sweat gland pores (diameter: 60-80 μm) and lipid matrix that fills a gap of 75 nm between dead corneocytes in the SC [39-41]. As the intact skin has more than one layer in humans, it is expected that ivy nanoparticles will have different diffusion activities in different layers. *Ex vivo *and *in vivo *experimental data supported that the SC has the most packaged properties and is not permeable to many chemicals and drugs [42]. A skin diffusion study indicated that the SC has a diffusion coefficient 10^3 ^times lower than the deeper viable layer for the same chemical [43]. Another study for nanoparticles in human skin also indicated that nanoparticles with a size of more than 20 nm rarely have a chance to penetrate through the SC layer [44]. Therefore, to understand ivy nanoparticle diffusion and penetration in human skin, it is essential to understand the diffusion process of these nanoparticles in the SC.

There are many papers dealing with the transport of nanoparticles through the SC layer of the skin in the current literature [15,31,45-48]. While the data vary depending on the experimental setup, it is generally agreed that the depth of penetration varies with the material properties of the nanoparticles, the size of individual particles, their shape, and other physicochemical factors [45]. Studies have suggested that sunscreens composed of TiO_2 _and ZnO nanoparticles do not pass into the upper layers of the SC. However, as mentioned earlier, these studies have only examined healthy adult skin models [11-14,49]. More realistically the skin to which the sunscreen will be applied has been damaged, either by prior sun exposure, or by a variety of other factors that damage the skin. We have previously discussed how damage to the skin and small particle size increase the depth to which nanoparticles will penetrate [15,16,29-31].

Despite the heterogeneous structure of the SC layer, in cases where penetration is concerned, the skin behaves as a homogeneous membrane and the diffusion law still holds [50-53]. To understand the dynamic activities of the ivy nanoparticles applied to the skin, Fick's Second Law is applied and described as the follows: (∂ϕ;/∂t) = D(∂^2^ϕ/∂χ^2^). The determination factor is the diffusion coefficient (D). This D is normally defined to be: D = k_B_T/6πηR. In the case of ivy nanoparticles, R is radius of ivy nanoparticles and is set at the value of 32.65 nm, η is the viscosity of the SC lipid matrix (0.02 kg/m s), k is the Boltzmann constant (1.38 × 10^-23 ^m^2 ^kg s^-2 ^K^-1^), and T is the absolute temperature for human skin (310.15 K). In this simplified model the surface properties of varying nanoparticles are ignored and only the radius of the nanoparticles effects diffusion. The SC layer, which is 20 μm deep, is lipophilic and acidic with variations in gender, anatomical sites, and environmental settings [54].

**Figure 5:**
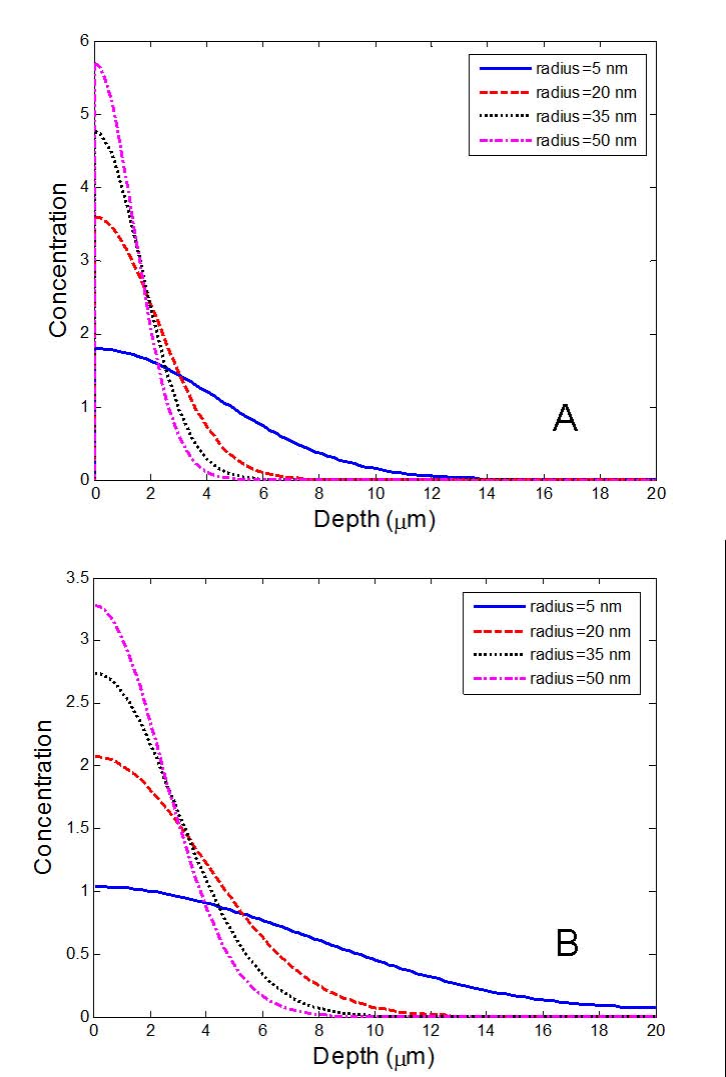
**Distribution of nanoparticles in SC layer of skin**. Computational simulation results for the distribution of nanoparticles in the SC layer of human skin 8 hours (A) and 20 hours (B) after application to the surface of the skin.

Based on the model and obtained parameters, the dynamics of nanoparticle diffusion in the SC layer of the skin was simulated. In Figure 5, the predicted distribution of different-sizes of nanoparticles after 8 and 20 hours of application is shown. While nanoparticles with a diameter less than 10 nm have a chance to reach into the bottom of the SC layer (Figure 5), nanoparticles over 40 nm can only reach 5-8 μm into the SC layer after 8 hours of application and 8-13 μm after 20 hours, as displayed in Figure 5. This agrees well with previous experimental studies about other nanoparticles within the same size range [55]. Considering that the normal period of exposure to sunlight in humans is less than 8 hours, and the diameter of the ivy nanoparticles is 65.3 nm, we expect that ivy nanoparticles can be used in cosmetics applications without a risk of penetration.

## Conclusions

The concern for the biosafety and health risk for the metal-based and engineered nanoparticles in sunscreens has led to the search for alternative replacement nanoparticles. In this study, naturally occurring ivy nanoparticles were investigated to replace TiO_2 _and ZnO that are currently widely used in sunscreen products. Based on experimental data, we have demonstrated that ivy nanoparticles have the potential levels of UV protection necessary to warrant further investigation for uses in cosmetics. The cell toxicity of ivy nanoparticles was next tested and it was determined that ivy nanoparticles exhibited much less toxicity than widely used TiO_2 _nanoparticles. Without obtaining the proper marker for experimental determination, a mathematical model was used to analyze the diffusion dynamics in the human skin, especially in the SC layer. Through this analysis, we found ivy nanoparticles with a diameter of 65.3 nm will not reach the bottom of SC layer in normal conditions for short periods of time after application. The biodegradability of these ivy nanoparticles further eliminates concerns regarding environmental contamination and in the case of entry into the body. All of the above studies demonstrated that naturally occurring ivy nanoparticles could be a promising alternative for UV protection in cosmetics, especially with concerns regarding the safety of metal-based nanoparticles. With increased dangers associated with more UV passing through the atmosphere [56], the need to protect human from skin cancer elicits the need for safe and effective UV protective agents. The promising application of these ivy nanoparticles thus provides a better chance to help protect people from UV radiation.

## Methods

### Ivy nanoparticle isolation

Juvenile *Hedera helix *shoots were grown in a greenhouse. The plant was originally taken naturally on the University of Tennessee, Knoxville campus that was transplanted to a pot in a greenhouse. Leaves were removed from the shoots and shoots were trimmed to 6 cm in length. Shoots were sterilized using 1.23% sodium hypochlorite (20% [v/v] commercial bleach) plus 0.05% Tween 20, shaken at 200 rpm for 20 min, and followed by washing three times with sterile water. Sterile shoots were then placed upright into Magenta GA7 boxes containing Murashige and Skoog (MS) medium and were grown at 24°C at 16:8 h photoperiod under 82 μmol m^-2^s^-1^irradiance. Aerial roots (rootlets) were produced after ca. 2 d, which were allowed to grow for an additional 2 days in MS medium to reach approximately 3 cm in length. Aerial roots were then excised from source plants and were transferred to Petri dishes containing MS medium until analysis was performed (between 3 days and 2 weeks). To isolate ivy nanoparticles, the tips of rootlets were homogenized in double-distilled water. After centrifugation to remove tissue residuals, the solution was dialysed overnight, then was loaded the BioSep-SEC-S 4000 size exclusion chromatography column and the elution fractions were collected for further analysis.

### Atomic force microscopy

The isolated ivy nanoparticle fractions were first air-dried overnight. The air-dried ivy samples were scanned for the existence of nanoparticles using an Agilent 5500 atomic force microscope (Agilent Technologies, Santa Clara, CA). The samples were imaged at room temperature (20°C) using Picoview™ in tapping mode, which minimizes sample distortion due to mechanical interactions between AFM tip and the surface. To further optimize imaging, the set point amplitude and the amplitude of the oscillating cantilever were adjusted to avoid excessive loading force applied to the samples. In this way, three-dimensional imaging of the surface morphology with very high lateral and vertical resolution has been obtained. The used tips are commercially available silicon probes TAP300Al^® ^(Budget Sensors, Sofia, Bulgaria) with a spring constant of 20-75 N/m, a resonant frequency of 300 ± 100 kHz, and a radius of curvature in less than 10 nm.

### Sample preparation

The calculation of isolated ivy nanoparticles was based on the number of the particles on the AFM images and the used volume to obtain these nanoparticles. To prepare the same concentration of TiO_2 _suspension, TiO_2 _particles with a diameter of 50 nm (99% purity) were purchased from Nanostructured & Amorphous Materials Inc. (Houston, TX). The particles were first ultrasonically dispersed in water at 5 W for 30 min, then were used for later UV-Vis study and cytotoxicity study.

### UV-Vis extinction

The UV-Vis extinction (absorption and scattering) spectra were measured using a Thermo Scientific Evolution 600 UV-Visible spectrophotometer (Thermo Fisher Scientific, Waltham, MA). The optical length of the quartz cuvette was 10 mm. The wavelength of the light started at 250 nm and stopped at 800 nm. Ivy nanoparticles at 4.92 μg/ml were measured for UV-Vis. The same concentration of TiO_2 _nanoparticles was measured for UV-Vis under the same conditions.

### Cytotoxicity study

HeLa cells were cultured in a DMEM (Mediatech Inc, Manassas, VA) solution supplemented with 10% heat-inactivated fetal bovine serum in a humidified incubator with an atmosphere of 5% CO_2 _in air at 37°C. The TiO_2 _or ivy nanoparticle aqueous suspension was added to DMEM solution supplemented with 10% fetal bovine serum to prepare a DMEM solution containing nanoparticles, which was used to investigate the cytotoxicity against HeLa cells. Negative controls consisted of DMEM with 10% fetal bovine serum, without the presence of nanoparticles. For apoptosis analysis, the cells were harvested 24 hours after addition of nanoparticles, fixed and stained with propidium iodide, then were analyzed using Bechman Coulter Episc XL (Bechman Coulter, Brea, CA) with a 488 nm argon laser.

### Nanoparticle degradation

Purified nanoparticles were sonicated at 5 W for 20 minutes to physically disperse the nanoparticles so that individual ones that could be analyzed. SDS was then added to the nanoparticle solution to make a final concentration of 0.5%. To the following, 50 μg/ml of Proteinase K was added. The solution was then incubated at 37°C from 15 minutes to 4 hours. Upon completion of the digestion, the sample was air-dried and imaged using AFM to determine if the nanoparticles were degraded. The control sample was prepared in the same way except that the Proteinase K was not added before incubation. In addition to examining enzymatic digestion with Proteinase K, the effects of varying temperatures were examined by incubation of the nanoparticles from 4-37°C. Similarly, the nanoparticles were added to RPMI for 24 hours to determine their stability in a typical cell culture media. All samples were then air-dried and imaged by AFM.

### Statistical analysis

To determine if there were significant differences in cytotoxicity among HeLa cells incubated without or with different nanoparticles, a Student's *t *test for comparisons of each pair with 95% confidence was carried out using JMP 8 statistical software.

## Competing interests

The authors declare that they have no competing interests.

## Authors' contributions

LX performed the majority of the experiments and wrote the manuscript with SCL and MZ. QL contributed with the characterization by spectrophotometry and helped with data analysis. MZ, ZZ, SCL and LX designed the overall project. MZ and SCL helped with the interpretation of data and revised the manuscript. All authors read and approved the manuscript.
